# Supplementary material for: Content-rich biological network constructed by mining PubMed abstracts
Source: BMC Bioinformatics. 2004 Oct 8;5:147. doi: 10.1186/1471-2105-5-147 (PMC528731; doi:10.1186/1471-2105-5-147)
Supplement: Additional File 2 — The original results of the above study (non-essential files are deleted to keep the file size under the limit set by BMC bioinformatics). [file 1471-2105-5-147-S2.bz2 › chilibotAdditionalFile2/dip10/494ID10187774E10657/html/TGFBR1_TGFB3.html]

 


 **TGFBR1** and **TGFB3** 
  
Found 25 abstracts in PubMed, retrieved 10.  
 

 What does Google say? 
 PDF only 
| .edu only 

---

**Interactive relationship** (e.g. stimulation, inhibition, etc)

**Non-interactive relationship** (e.g. studied together, co-existance, homology, etc.)

- We therefore studied the expression of UPA, UPAR, IGF2R, ALK5  [ **TGFBR1** ] , TGFBR2, TGFBR3, ENG, ALK1, TGFB1, TGFB2, and  **TGFB3**  in a series of 14 pancreatic carcinoma cell lines.  Ref: 11408925 Int J Oncol, 2001
- METHODS AND RESULTS The spatial relationships between TGF beta1, TGF beta3  [ **TGFB3** ] , ALK 5  [ **TGFBR1** ] , and TbetaR II expression were compared in aortic segments from 21 subjects.  Ref: 10359732 Circulation, 1999
- Boston minipigs, treated with tranilast or vehicle, were subjected to endoluminal stenting, and the expression of TGF beta1 and TGF beta3  [ **TGFB3** ] , the expression of their signaling receptors ALK 5  [ **TGFBR1** ]  and TbetaR II, leukocyte numbers around the stent struts, and neointima development were assessed over 28 days.  Ref: 12067902 Arterioscler Thromb Vasc Biol, 2002
- Although neither TGF beta2 nor TGF beta3  [ **TGFB3** ]  expression was affected by hypoxia, the TGF beta type I receptor  [ **TGFBR1** ]  was substantially upregulated within 6 h.  Ref: 11421617 J Surg Res, 2001
